# The unit ratio-extended Weibull family and the dropout rate in Brazilian undergraduate courses

**DOI:** 10.1371/journal.pone.0290885

**Published:** 2023-11-16

**Authors:** Fernando A. Peña-Ramírez, Renata R. Guerra, Charles Peixoto Mafalda

**Affiliations:** 1 Departamento de Estadística, Universidad Nacional de Colombia, Bogotá, Colombia; 2 Departamento de Estatística, Universidade Federal de Santa Maria, Santa Maria, RS, Brazil; 3 Departamento de Estatística, Universidade Federal de Pernambuco, Recife, PE, Brazil; Cairo University, EGYPT

## Abstract

We propose a new family of distributions, so-called the unit ratio-extended Weibull family (UREW). It is derived from ratio transformation in an extended Weibull random variable. The use of this transformation is a novelty of the work since it has been less explored than the exponential and has not yet been studied within the extended Weibull class. Moreover, we offer a valuable alternative to model double-bounded variables on the unit interval. Five UREW special models are studied in detail, namely the: i) unit ratio-Gompertz; ii) unit ratio-Burr XII; iii) unit ratio-Lomax; v) unit ratio-Rayleigh, and vi) unit ratio-Weibull distributions. We propose a quantile-parameterization for the new family. The maximum likelihood estimators (MLEs) are presented. A Monte Carlo study is performed to evaluate the behavior of the MLEs of unit ratio-Gompertz and unit ratio-Rayleigh distributions. This last model has closed-form and approximately unbiased MLE for small sample sizes. Further, the UREW submodels are adjusted to the dropout rate in Brazilian undergraduate courses. We focus on the areas of civil engineering, economics, computer sciences, and control engineering. The applications show that the new family is suitable for modeling educational data and may provide effective alternatives compared to other usual unit models, such as the Beta, Kumaraswamy, and unit gamma distributions. They can also outperform some recent contributions in the unit distribution literature. Thus, the UREW family can provide competitive alternatives when those models are unsuitable.

## 1 Introduction

The formulation of new generalized classes of probability distributions is a topic that has received a great deal of attention in recent years, particularly when it comes to positive data [1]. To mention a few, we refer the reader to [2–4] as extensions of the Weibull distribution and [5, 6] for Nadarajah-Haghighi generalizations. Most of these works are introduced aiming to furnish more flexible distributions regarding shape densities and hazard rates. However, there is much to be done when considering random variables supported in the unit interval. We can cite the beta and Kumaraswamy [7] (KW) distributions as classical unit models In this respect.

Motivated by the increasing interest in modeling bounded data, other unit distributions have been introduced and are available in the literature. Some of these advances are, for instance, the unit gamma [8] (UG), simplex [9], CDF-quantile [10], unit Birnbaum-Saunders [11, 12] (UBS), unit Weibull [13] (UW), unit extended Weibull [14], complementary unit extended Weibull [14], unit Gompertz [15], unit Burr XII [16, 17], reflected unit Burr XII [18], unit generalized half normal [19], bounded odd inverse Pareto exponential [20], Modified Kumaraswamy [21], unit-sinh-normal [22], log-Bilai [23] and log-weighted exponential [24] distributions. This interest is due to several natural and anthropogenic phenomena which are bounded in a certain interval [12]. The list of double-bounded random variables may include the proportion of chemical components in different substances [25], vote proportions [12, 26], relative air humidity [27], well-being indicators [14], mortality rates [18, 28], loss given default [29] among several other indexes, indicators, ratios, and rates. Nevertheless, some situations may require other alternatives to model heavy tails and asymmetric proportion data where current models have limitations.

In this context, we introduce the so-called unit ratio-extended Weibull (UREW) family of distributions, which is built upon the ratio transformation in the extended Weibull [30] (EW) class. The most common method to derive those unit distributions is applying the exponential transformation in positive random variables. The use of ratio transformation is a novelty of this work since it has been less explored and has not yet been studied within the EW class. One advantage of introducing the UREW is that some special models can produce N-shaped, U-shaped, and unimodal density shapes. These features make the proposed family quite attractive for educational modeling and addressing real-life problems involving asymmetric and heavy-tailed double-bounded indicators. The N-shaped behavior, for example, is not assumed by the classical beta and KW distributions but can be accommodated by some UREW special cases. We conduct shape analysis and provide density plots on the proposed models to illustrate these characteristics.

Our main contribution lies in offering a valuable alternative to model double-bounded variables in the unit interval. Moreover, we present at least four contributions achieved by pioneering the UREW class. First, the new family has more than twenty special models that may provide a source of alternatives to deal with rates and proportions, among other random variables in the unit domain. The second contribution is to provide a quantile parametrization for the new family. This framework is useful since the quantiles are outlier-resistant location measures and have a more intuitive interpretation than the original parametrization. The third contribution is related to parameter estimation under the maximum likelihood approach. As illustrated in Section 4, some one-parameter UREW special cases present its maximum likelihood estimator (MLE) in closed form.

Finally, the fourth contribution to formulating the UREW family is its applicability for modeling educational indicators. This type of data has motivated the proposal of several unit distributions. It is the case of [31–33], which examines indicators related to educational attainment percentage and school living conditions across various countries. We can also cite [14] for analyzing literacy rates in Brazilian and Colombian municipalities and [17] for modeling the dropout rate of Brazilian undergraduate animal science courses. However, it should be noted that, to the author’s knowledge, there remains a significant gap in the available information regarding the phenomenon of first-year, or freshman, student dropout. This paper’s motivating data sets concern the first-year dropout rate in Brazilian undergraduate courses. We analyze this outcome for civil engineering, economics, computer sciences, and control engineering courses. The four data sets are positive-skewed, and we observe a short amount of courses with a dropout rate smaller than 17%. This feature should be common for these kinds of data. When analyzing higher education institutions, academic programs with low dropout rates tend to receive higher quality ratings [34]. In addition, this measure is seen as an indicator of institutional excellence and performance [35]. Therefore, our proposals have the advantage of providing consistently better fits than classical beta and KW distributions when modeling the dropout rate in Brazilian undergraduate courses (see Section 6). As illustrated in the applications, they can also outperform some recent contributions in the distribution literature, such as the UBS, UW, and CUW distributions. All analysis in this paper is carried out using R programming language. The computational codes and data sets used to obtain the plots, simulations, and application results are made available on a GitHub repository (Computer codes available at https://github.com/penaramirez/UREW).

The rest of the paper is organized as follows. Section 2 presents the theoretical background and defines the new family of unit distributions. Some UREW special cases are presented in Section 3. Section 4 focuses on inferential procedures based on the maximum likelihood method. We present results for all family members and derive expressions for the MLEs of some special models. Section 5 discusses simulation studies’ results to assess the performance of the point and asymptotic interval estimators. Section 6 illustrates our proposed family’s relevance in educational data, specifically about the first-year dropout rate in some Brazilian undergraduate courses. The final remarks are presented in Section 7.

## 2 The unit ratio-extended Weibull family of distributions

This section presents the theoretical background and defines the proposed family from a ratio transformation in the EW class of distributions. Let *X* be a random variable on the EW class, and denote X∼EW(α,ξ). The probability density function (pdf) of *X* is given by

$$\begin{array}{c} g\left(x\right)=\alpha h\left(x;\xi \right)\;\text{exp}\;[-\alpha H(x;\xi )], \end{array}$$ (1)

where *x* > 0, *α* > 0, *H*(*x*; ***ξ***) is a non-negative monotonically increasing function which depends on the parameter vector ***ξ***, and *h*(*x*; ***ξ***) is the derivative of *H*(*x*; ***ξ***) with respect to *x*. For each formulation of *H*(*x*; ***ξ***), different EW special models result. Thus, several well-known distributions can be obtained depending on the choice of this function. Table 1 presents twenty alternatives for *H*(*x*; ***ξ***), their corresponding derivatives, and inverse functions. Further details on this family and some generalizations to examine non-negative data are given by [36–38].

**Table 1:** Some EW special models and their corresponding *H*(*x*; *ξ*), *H*^−1^(*x*; *ξ*) and *h*(*x*; *ξ*) functions.

Distribution	*H*(*x*; *ξ*)	*H*^−1^(*x*; *ξ*)	*h*(*x*; *ξ*)	*α*	***ξ***
Additive Weibull	$\begin{array}{c} {\left(x/\beta_{1}\right)}^{\alpha_{1}}+{\left(x/\beta_{2}\right)}^{\alpha_{2}} \end{array}$	no closed-form	$\begin{array}{cc} & \left(\alpha_{1}/\beta_{1}\right){\left(x/\beta_{1}\right)}^{\alpha_{1}-1}\\ & +\left(\alpha_{2}/\beta_{2}\right){\left(x/\beta_{2}\right)}^{\alpha_{2}-1} \end{array}$	1	$\begin{array}{cc} & [\alpha_{1},\alpha_{2},\\ & \beta_{1},\beta_{2}] \end{array}$
Burr XII	log(1 − *x*^*β*^)	[1 − exp(*x*)]^1/*β*^	*βx*^*β*−1^/(1 + *x*^*β*^)	*α*	*β*
Chen	exp(*x*^*b*^) − 1	[log(*x* + 1)]^1^/*b*	*bx*^*b*−1^ exp(*x*^*b*^)	*α*	*b*
Exponential	*x*	*x*	1	*α*	∅
Exponential power	exp[(λ*x*)^*β*^] − 1	λ^−1^[log(*x* + 1)]^1/*β*^	*β*λ exp[(λ*x*)^*β*^](λ*x*)^*β*−1^	1	[λ, *β*]
Flexible Weibull	exp(λ*x* − *β*/*x*)	(*)	exp(λ*x* − *β*/*x*)(λ + *β*/*x*^2^)	1	[λ, *β*]
Fréchet	*x* ^−*y*^	*x* ^−1/*y*^	−*yx*^−(*y*+1)^	*α*	*y*
Gompertz (*x* > 0)	*β*^−1^[exp(*βx*) − 1]	*β*^−1^ log(*βx* + 1)	exp(*βx*)	*α*	*β*
Linear Failure rate	*ax* + *bx*^2^/2	no closed-form	*a* + *bx*	1	[*a*, *b*]
Log-logistic	log(1 + *x*^*c*^)	[1 − exp(*x*)]^1/*c*^	*cx*^*c*−1^/(1 + *x*^*c*^)	1	*c*
Lomax	log(1 + *x*/*β*)	*β*[exp(*x*) − 1]	1/(*β* + *x*)	*α*	*β*
Modified Weibull	*x*^*y*^ exp(λx)	(*)	*x*^*y*−1^ exp(λx)(*y* + λx)	*α*	[*y*, λ]
Pareto (*x* > *k*)	log(*x*/*k*)	*k* exp(*x*)	1/*x*	*α*	*k*
Power generalized Weibull	${\left[1+{\left(x/\beta \right)}^{\alpha_{1}}\right]}^{\theta }-1$	$\beta {\left[{\left(x+1\right)}^{1/\theta }-1\right]}^{1/\alpha_{1}}$	$\begin{array}{cc} & \left(\theta \alpha_{1}/\beta \right){\left[1+{\left(x/\beta \right)}^{\alpha_{1}}\right]}^{\theta -1}\\ & {\left(x/\beta \right)}^{\alpha_{1}} \end{array}$	1	[*α*_1_, *β*, *θ*]
Rayleigh	*x* ^2^	*x* ^1/2^	2*x*	*α*	∅
Weibull	*x* ^*y*^	*x* ^1/*y*^	*yx* ^*y*−1^	*α*	*y*

The EW cumulative distribution function (cdf) and quantile function (qf) are given by

$$\begin{array}{c} G\left(x\right)=1-\text{exp}[-\alpha H(x;\xi )], \end{array}$$

and

$$\begin{array}{c} Q\left(u\right)=H^{-1}[-\frac{1}{\alpha }\;\text{log}\;\left(1-u\right)], \end{array}$$

respectively, where *H*^−1^(⋅; ***ξ***) is the inverse function of *H*(⋅; ***ξ***).

We define the UREW class of distribution by considering the ratio transformation *Y* = *X*/(1 + *X*), where X∼EW(α,ξ). Hereafter, we denote *Y* as a UREW random variable, which has cdf

$$\begin{array}{c} F_{Y}\left(y\right)=1-\text{exp}[-\alpha H(\frac{y}{1-y};\xi )], \end{array}$$ (2)

where 0 < *y* < 1, *α* > 0, and ***ξ*** is the parameter vector associated to the *H*(⋅; ***ξ***) function.

Thus, the pdf and qf of the proposed family are

$$f_{Y}\left(y\right)=\alpha \;{\left(1-y\right)}^{-2}\;h(\frac{y}{1-y};\xi )\;\text{exp}\;[-\alpha H(\frac{y}{1-y};\xi )],$$

and

$$\begin{array}{c} Q_{Y}\left(u\right)=\frac{H^{-1}[-\alpha^{-1}\;\text{log}\left(1-u\right);\xi ]}{1+H^{-1}[-\alpha^{-1}\;\text{log}\left(1-u\right);\xi ]}, \end{array}$$

respectively. The proposition below refers to a quantile-based parametrization for the UREW family. Analogous frameworks can be found in other unit models recently introduced. See [14, 39, 40] for median-based parametrizations and [41] for a quantile-based example.

**Proposition 1**. *Let Y be a* UREW *random variable, then its cdf can be rewritten as*

$$\begin{array}{c} F_{Y}\left(y\right)=1-{\left(1-\tau \right)}^{H(\frac{y}{1-y};\xi )/H(\frac{q(\tau )}{1-q(\tau )};\xi )},\;y\in \left(0,1\right), \end{array}$$ (3)

*where q*(*τ*) ∈ (0, 1) *is a location parameter which corresponds to the τth quantile of Y*, ***ξ*** *is the parameter vector associated with H*(⋅; ***ξ***), *and τ is assumed as known*.

*Proof*. The result in Eq (3) holds by replacing $\alpha =\text{log}(1-\tau {)}^{-1}/H(\frac{q(\tau )}{1-q(\tau )};\xi )$ in (2). Hence, the qf *Y* can be rewritten as

$$\begin{array}{c} Q_{Y}\left(u\right)=\frac{H^{-1}[H(\frac{q(\tau )}{1-q(\tau )};\xi )\frac{\text{log}(1-u)}{\text{log}(1-\tau )};\xi ]}{1+H^{-1}[H(\frac{q(\tau )}{1-q(\tau )};\xi )\frac{\text{log}(1-u)}{\text{log}(1-\tau )};\xi ]}. \end{array}$$

Setting *u* = *τ* in the above equation, we obtaing that *Q*_*Y*_(*τ*) = *q*(*τ*), which concludes the proof.

Under the quantile parametrization, the UREW pdf can be written as

$$\begin{array}{c} f_{Y}\left(y\right)=\frac{\text{log}[{\left(1-\tau \right)}^{-1}]}{{\left(1-y\right)}^{2}H(\frac{q(\tau )}{1-q(\tau )};\xi )}\;h(\frac{y}{1-y};\xi )\;{\left(1-\tau \right)}^{H(\frac{y}{1-y};\xi )/H(\frac{q(\tau )}{1-q(\tau )};\xi )}. \end{array}$$ (4)

## 3 Some UREW special cases

Several well-established statistical models are special cases in the EW family. They can be considered baseline models in the UREW family by replacing their corresponding *H*(⋅; ***ξ***) functions in the cdf (2). Here, we give further details on five of those models, namely: the unit ratio-Gompertz (URG), unit ratio-Burr XII (URBXII), unit ratio-Lomax (URL), unit ratio-Weibull (URW), and unit ratio-Rayleigh (URR) distributions. These models are introduced using the quantile-parametrization given in Proposition 1. The *H*(⋅; ***ξ***) functions of these and several other models members of the UREW family can be consulted in Table 1.

### 3.1 The unit ratio-Gompertz distribution

**Fig 1:**
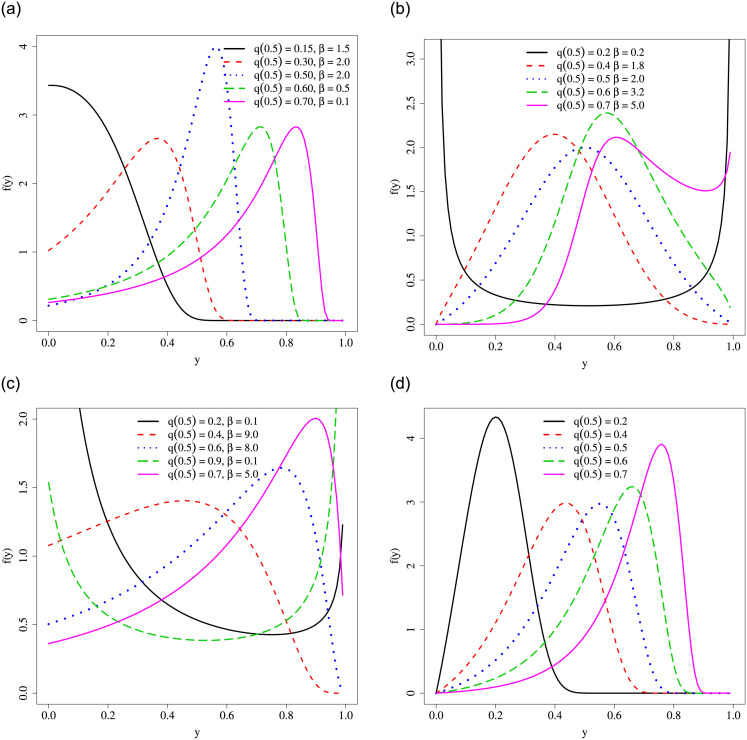
Density plots for some UREW special models. (a) URG, (b) URBXII, (c) URL, (d) URR.

The URG distribution is obtained considering the Gompertz as a baseline model in the UREW family. Thus, by taking *H*(*x*; ***ξ***) = exp{*βx*} − 1 in (2), the URG cdf can be written as

$$\begin{array}{c} F_{URG}\left(y\right)=1-{\left(1-\tau \right)}^{\left(\text{exp}\{\frac{\beta y}{1-y}\}-1)/(\text{exp}\{\frac{\beta \;\mu }{1-\mu }\}-1\right)}, \end{array}$$

where *y* ∈ (0, 1), *β* > 0 is a shape parameter, and *μ* ∈ (0, 1) is the *τ*th quantile parameter. The corresponding pdf, qf, and hazard rate function (hrf) are

$$\begin{array}{cc} f_{URG}\left(y\right) & =\frac{\beta \;\text{log}[{\left(1-\tau \right)}^{-1}]\;\text{exp}\{\frac{\beta y}{1-y}\}}{{\left(1-y\right)}^{2}[\text{exp}\{\frac{\beta \;\mu }{1-\mu }\}-1]}\;\;\\ & \times {\left(1-\tau \right)}^{\left(\text{exp}\{\frac{\beta y}{1-y}\}-1)/(\text{exp}\{\frac{\beta \;\mu }{1-\mu }\}-1\right)}, \end{array}$$ (5)

$$\begin{array}{c} Q_{URG}\left(u\right)=\frac{\text{log}\{\frac{\text{log}(1-u)}{\text{log}(1-\tau )}\;[\text{exp}(\frac{\beta \;\mu }{1-\mu })-1]+1\}}{\beta \;+\text{log}\{\frac{\text{log}(1-u)}{\text{log}(1-\tau )}\;[\text{exp}(\frac{\beta \;\mu }{1-\mu })-1]+1\}}, \end{array}$$ (6)

and

$$h_{URG}\left(y\right)=\frac{\beta \;\text{log}[{\left(1-\tau \right)}^{-1}]\;\text{exp}\{\frac{\beta y}{1-y}\}}{{\left(1-y\right)}^{2}[\text{exp}\{\frac{\beta \;\mu }{1-\mu }\}-1]},$$

respectively. Fig 1(a) illustrates the URG pdf shapes for several combinations of *μ* and *β*, with *τ* = 0.5.

### 3.2 The unit ratio-Burr XII distribution

The URBXII distribution is obtained considering the Burr XII as a baseline model in the UREW family. Thus, by taking *H*(*x*; ***ξ***) = log[1 + *x*^*β*^] in (2), and after simplification, the URBXII cdf reduces to

$$\begin{array}{c} F_{URBXII}\left(y\right)=1-{\left[1+\frac{y^{\beta }}{{\left(1-y\right)}^{\beta }}\right]}^{\text{log}(1-\tau )/\text{log}\{1+q{\left(\tau \right)}^{\beta }{\left[1-q\left(\tau \right)\right]}^{-\beta }\}} \end{array}$$

where *y* ∈ (0, 1), and *μ* ∈ (0, 1) is the *τ*th quantile parameter. The corresponding pdf, qf, and hrf are

$$\begin{array}{cc} f_{URBXII}\left(y\right) & =\frac{\beta y^{\beta -1}\;\text{log}[{\left(1-\tau \right)}^{-1}]}{{\left(1-y\right)}^{\beta +1}\;\text{log}\{1+q{\left(\tau \right)}^{\beta }{\left[1-q\left(\tau \right)\right]}^{-\beta }\}}\\ & \times {\left[1+\frac{y^{\beta }}{{\left(1-y\right)}^{\beta }}\right]}^{\text{log}(1-\tau )/\text{log}\{1+q{\left(\tau \right)}^{\beta }\left[1-q{\left(\tau \right)}^{-\beta }\right]\}-1}, \end{array}$$ (7)

$$\begin{array}{c} Q_{URBXII}\left(u\right)=\frac{{\left[{\left(1-u\right)}^{\text{log}\{1+q{\left(\tau \right)}^{\beta }{\left[1-q\left(\tau \right)\right]}^{-\beta }\}\text{log}(1-\tau )}-1\right]}^{1/\beta }}{1+{\left[{\left(1-u\right)}^{\text{log}\{1+q{\left(\tau \right)}^{\beta }{\left[1-q\left(\tau \right)\right]}^{-\beta }\}\text{log}(1-\tau )}-1\right]}^{1/\beta }}, \end{array}$$ (8)

and

$$h_{URBXII}\left(y\right)=\frac{\beta y^{\beta -1}\;\text{log}[{\left(1-\tau \right)}^{-1}]}{{\left(1-y\right)}^{\beta +1}\;\text{log}\{1+q{\left(\tau \right)}^{\beta }{\left[1-q\left(\tau \right)\right]}^{-\beta }\}},$$

respectively. Fig 1(b) illustrates the URBXII pdf shapes for several combinations of *μ* and *τ* = 0.5. This plot illustrates the flexibility of the URBXII distribution. It can have N-shaped, U-shaped, and unimodal density shapes, being able to fit asymmetric and heavy-tailed double-bounded data.

### 3.3 The unit ratio-Lomax distribution

The URL distribution is obtained considering the Lomax as a baseline model in the UREW family. Thus, by taking *H*(*x*; ***ξ***) = log[1 + *x*^*β*^] in (2), and after simplificaion, the URL cdf reduces to

$$\begin{array}{c} F_{URL}\left(y\right)=1-{\left[1+\frac{y}{\beta (1-y)}\right]}^{\text{log}\left(1-\tau \right)/\text{log}[1+\frac{\mu }{\beta (1-\mu )}]} \end{array}$$

where *y* ∈ (0, 1), *β* > 0 is a shape parameter, and *μ* ∈ (0, 1) is the *τ*th quantile parameter. The corresponding pdf, qf, and hrf are

$$\begin{array}{cc} f_{URL}\left(y\right) & =\frac{\text{log}[{\left(1-\tau \right)}^{-1}]}{\left(1-y\right)[\beta (1-y)+y]\text{log}[1+\frac{\mu }{\beta (1-\mu )}]}\\ & \times {\left[1+\frac{y}{\beta (1-y)}\right]}^{\text{log}\left(1-\tau \right)/\text{log}[1+\frac{\mu }{\beta (1-\mu )}]}, \end{array}$$ (9)

$$Q_{URL}\left(u\right)=\frac{\beta {\left(1-u\right)}^{\text{log}[1+\frac{\mu }{\beta (1-\mu )}]/\text{log}\left(1-\tau \right)}-\beta }{1+\beta {\left(1-u\right)}^{\text{log}[1+\frac{\mu }{\beta (1-\mu )}]/\text{log}\left(1-\tau \right)}-\beta },$$

and

$$\begin{array}{c} h_{URL}\left(y\right)=\frac{\text{log}[{\left(1-\tau \right)}^{-1}]}{\left(1-y\right)[\beta (1-y)+y]\text{log}[1+\frac{\mu }{\beta (1-\mu )}]}, \end{array}$$

respectively. Fig 1(c) illustrates the URL pdf shapes for several combinations of *μ* and *β*, with *τ* = 0.5.

### 3.4 The unit ratio-Weibull and unit ratio-Rayleigh distributions

The URW distribution is obtained considering the Weibull as baseline model in the UREW family. By taking *H*(*x*; ***ξ***) = *x*^*β*^ in (2), the URW can be written as

$$\begin{array}{c} F_{URW}\left(y\right)=1-{\left(1-\tau \right)}^{y^{\beta }\;{\left(1-\mu \right)}^{\beta }/\left[\mu^{\beta }\;{\left(1-y\right)}^{\beta }\right]}, \end{array}$$

where *y* ∈ (0, 1), *β* > 0 is a shape parameter, and *μ* ∈ (0, 1) is the *τ*th quantile parameter. The corresponding pdf, qf, and hrf are

$$\begin{array}{c} f_{URW}\left(y\right)=\frac{\beta \;y^{\beta -1}\;{\left(1-\mu \right)}^{\beta }}{\mu^{\beta }\;{\left(1-y\right)}^{\beta +1}}[\text{log}{\left(1-\tau \right)}^{-1}]\;{\left(1-\tau \right)}^{y^{\beta }\;{\left(1-\mu \right)}^{\beta }/\left[\mu^{\beta }\;{\left(1-y\right)}^{\beta }\right]}, \end{array}$$ (10)

$$\begin{array}{c} Q_{URW}\left(u\right)=\frac{{\left[\frac{\mu^{\beta }\;\text{log}\left(1-u\right)}{{\left(1-\mu \right)}^{\beta }\;\text{log}(1-\tau )}\right]}^{1/\beta }}{1+{\left[\frac{\mu^{\beta }\;\text{log}\left(1-u\right)}{{\left(1-\mu \right)}^{\beta }\;\text{log}(1-\tau )}\right]}^{1/\beta }}, \end{array}$$

and

$$\begin{array}{c} h_{URW}\left(y\right)=\frac{\beta \;y^{\beta -1}\;{\left(1-\mu \right)}^{\beta }}{\mu^{\beta }\;{\left(1-y\right)}^{\beta +1}}[\text{log}{\left(1-\tau \right)}^{-1}], \end{array}$$

respectively. For *β* = 2, the URW reduces to the URR distribution, which is also new. The URR is a one-parameter model obtained considering the Rayleigh as a baseline model in the UREW family. Its pdf is given by

$$\begin{array}{c} f_{URR}\left(y\right)=\frac{2\;y\;{\left(1-\mu \right)}^{2}}{\mu^{2}\;{\left(1-y\right)}^{3}}\text{log}{\left(1-\tau \right)}^{-1}\;{\left(1-\tau \right)}^{y^{2}\;{\left(1-\mu \right)}^{2}/\left[\mu^{2}\;{\left(1-y\right)}^{2}\right]}. \end{array}$$ (11)

Fig 1(d) illustrates the URR pdf shapes for several combinations of *μ* and *τ* = 0.5. It shows that the URR distribution presents unimodal density shape, accomodating left and right-skewed data in the unit interval.

## 4 Maximum likelihood estimation

Here, we consider estimation of the parameters of the UREW family by the maximum likelihood (ML) method. The log-likelihood for a random sample *y*_1_, … *y*_*n*_ from (4), based on parameter vetor ***θ*** = (*μ*, ***ξ***^⊤^)^⊤^, is

$$\begin{array}{c} \ell (\theta |y_{1},\ldots ,y_{n})=n\;\text{log}[\text{log}{\left(1-\tau \right)}^{-1}]-n\;\text{log}[H(\frac{\mu }{1-\mu };\xi )]\\ -2\;\sum_{i=1}^{n}\text{log}\left(1-y_{i}\right)+\sum_{i=1}^{n}\text{log}[h(\frac{y_{i}}{1-y_{i}};\xi )]-\;\frac{\text{log}{\left(1-\tau \right)}^{-1}}{H(\frac{\mu }{1-\mu };\xi )}\;\sum_{i=1}^{n}H(\frac{y_{i}}{1-y_{i}};\xi ). \end{array}$$ (12)

The components of the score vector ***U***(***θ***) = [*U*_*μ*_, *U*_***ξ***_]^⊤^, are

$$\begin{array}{c} U_{\mu }=\frac{h(\frac{\mu }{1-\mu };\xi )}{{\left(1-\mu \right)}^{2}H(\frac{\mu }{1-\mu };\xi )}[-n\;+\frac{\text{log}{\left(1-\tau \right)}^{-1}}{H(\frac{\mu }{1-\mu };\xi )}\sum_{i=1}^{n}H(\frac{y_{i}}{1-y_{i}};\xi )] \end{array}$$

e

$$\begin{array}{cc} U_{\xi } & =\frac{\partial \ell }{\partial \xi }=\sum_{i=1}^{N}\frac{1}{h(\frac{y_{i}}{1-y_{i}};\xi )}\frac{\partial \ell }{\partial \xi }\;\;h(\frac{y_{i}}{1-y_{i}};\xi )-n\;\frac{h(\frac{\mu }{1-\mu };\xi )}{H(\frac{\mu }{1-\mu };\xi )}-\frac{\text{log}{\left(1-\tau \right)}^{-1}}{H(\frac{\mu }{1-\mu };\xi )}\\ & \times \sum_{i=1}^{n}\frac{\partial \ell }{\partial \xi }\;h(\frac{y_{i}}{1-y_{i}};\xi )+\frac{\text{log}{\left(1-\tau \right)}^{-1}\;h(\frac{\mu }{1-\mu };\xi )}{{\left[H(\frac{\mu }{1-\mu };\xi )\right]}^{2}}\;\sum_{i=1}^{n}\frac{\partial \ell }{\partial \xi }\;H(\frac{y_{i}}{1-y_{i}};\xi ). \end{array}$$

For fixed values of ***ξ***, it is possible to obtain a closed-form for the MLE of the *μ*. By setting *U*_*μ*_ = 0 and solving for *μ*, we have

$$\begin{array}{c} \hat{\mu }\left(\hat{\xi }\right)=\frac{H^{-1}[\frac{1}{n}\text{log}{\left(1-\tau \right)}^{-1}\sum_{i=1}^{n}\frac{y_{i}}{1-y_{i}}\;;\xi ]}{1+\;H^{-1}[\frac{1}{n}\text{log}{\left(1-\tau \right)}^{-1}\sum_{i=1}^{n}\frac{y_{i}}{1-y_{i}}\;;\xi ]}. \end{array}$$

Therefore, obtaing the EMV of *μ* in closed-form is possible when ***ξ*** = ∅. Otherwise, to get the MLEs of the parameters *μ* and ***ξ***, it is necessary to use some iterative procedures such as Newton-Raphson type algorithms to maximize (12).

We can construct approximate confidence intervals for ***θ*** based on the asymptotic normality property. Under standard regularity conditions, the asymptotic distribution of $\hat{\theta }-\theta$ can be approximated by the multivariate normal $N(0,{J(\hat{\theta })}^{-1})$ distribution, where ${J(\hat{\theta })}^{-1}=-\partial \ell \left(\theta \right)/\partial \theta \partial \theta^{⊤}{|}_{\theta =\hat{\theta }}$ is the observed information matrix. Thus, the asymptotic 100(1 − *η*)% confidence intervals of ***θ*** are given by

$$\begin{array}{c} \hat{\theta }\pm z_{\eta /2}\times {\left[\hat{\text{var}}\;\left(\hat{\theta }\right)\right]}^{1/2}, \end{array}$$

where *z*_*η*/2_ is the quantile *η*/2 of the standard normal distribution, and $\hat{\text{var}}\;\left(\hat{\theta }\right)=\text{diag}\{{J(\hat{\theta })}^{-1}\}$. In what follows, we present the likelihood estimation of some special cases of the UREW family.

### 4.1 MLE for the URR(μ) distribution

Let *y*_1_, …, *y*_*n*_ be a random sample of size *n* from the URR(μ) distribution. The log-likelihood function is

$$\begin{array}{c} \ell (\mu |y_{1},\ldots ,y_{n})=n\;\text{log}\;\left(2\right)+\sum_{i=1}^{n}\text{log}\left(y_{i}\right)+n\;\text{log}\left[{\left(1-\mu \right)}^{2}\right]+n\;\text{log}[\text{log}{\left(1-\tau \right)}^{-1}]\\ -2n\;\text{log}\left(\mu \right)-3\;\sum_{i=1}^{n}\text{log}\left(1-y_{i}\right)-\frac{\text{log}{\left(1-\tau \right)}^{-1}{\left(1-\mu \right)}^{2}}{\mu^{2}}\;\sum_{i=1}^{n}\frac{y_{i}^{2}}{{\left(1-y_{i}\right)}^{2}}. \end{array}$$

The escore function *U*_*μ*_ is

$$\begin{array}{c} U_{\mu }=\frac{\partial \ell }{\partial \mu }=\frac{2}{\mu (1-\mu )}[-n+\frac{{\left(1-\mu \right)}^{2}\;\text{log}\;{\left(1-\tau \right)}^{-1}}{\mu^{2}}\sum_{i=1}^{n}\frac{y_{i}^{2}}{{\left(1-y_{i}\right)}^{2}}]. \end{array}$$ (13)

By setting *U*_*μ*_ = 0 and solving for *μ*, we have the EMV of *μ* as

$$\begin{array}{c} \hat{\mu }=\frac{\sqrt{\text{log}{\left(1-\tau \right)}^{-1}\sum_{i=1}^{n}\frac{y_{i}^{2}}{{\left(1-y_{i}\right)}^{2}}}}{\sqrt{n}+\sqrt{\text{log}{\left(1-\tau \right)}^{-1}\sum_{i=1}^{n}\frac{y_{i}^{2}}{{\left(1-y_{i}\right)}^{2}}}}, \end{array}$$ (14)

and the Fisher’s observed information is computed as

$$\begin{array}{c} I\left(\mu \right)=\frac{\partial^{2}\ell }{\partial \mu^{2}}=\frac{2}{\mu^{4}}[\frac{n\mu^{2}\left(1-2\mu \right)}{{\left(1-\mu \right)}^{2}}+\left(2\mu -3\right)\;\text{log}\;{\left(1-\tau \right)}^{-1}\sum_{i=1}^{n}\frac{y_{i}^{2}}{{\left(1-y_{i}\right)}^{2}}]. \end{array}$$ (15)

The conditions for the maximum value of the function *ℓ*(*μ*|*y*_1_, …, *y*_*n*_) require that $I(\hat{\mu })<0$. This is easily observed by substituting (14) into (15), where it is verified that

$$\begin{array}{c} I\left(\hat{\mu }\right)=-\frac{4(\sqrt{n}+\sqrt{\text{log}{\left(1-\tau \right)}^{-1}\sum_{i=1}^{n}\frac{y_{i}^{2}}{{\left(1-y_{i}\right)}^{2}}}{)}^{4}}{\text{log}{\left(1-\tau \right)}^{-1}\sum_{i=1}^{n}\frac{y_{i}^{2}}{{\left(1-y_{i}\right)}^{2}}}<0. \end{array}$$

### 4.2 MLE for the URG(β,μ) distribution

Let *y*_1_, …, *y*_*n*_ be a random sample of size *n* from the URG distribution with parameter vector ***θ*** = (*β*, *μ*)^⊤^. The log-likelihood function is

$$\begin{array}{c} \ell (\theta |y_{1},\ldots ,y_{n})=n\;\text{log}[\text{log}{\left(1-\tau \right)}^{-1}]-n\;\text{log}\left(\beta \right)-\frac{n\beta \mu }{1-\mu }+\frac{\text{log}[\text{log}{\left(1-\tau \right)}^{-1}]}{\text{exp}\{\frac{\beta \mu }{1-\mu }\}-1}\\ \times [\sum_{i=1}^{n}\text{exp}\{\frac{\beta y_{i}}{1-y_{i}}\}+n]-\sum_{i=1}^{n}\text{log}[{\left(1-y_{i}\right)}^{2}\;(\text{exp}\{\frac{\beta \;\mu }{1-\mu }\}-1)]. \end{array}$$ (16)

The components of the score vector *U*_***θ***_ = (*U*_*β*_, *U*_*μ*_)^⊤^ are

$$\begin{array}{cc} U_{\beta } & =\frac{\partial \ell }{\partial \beta }=\frac{n\mu }{1-\mu }-n\frac{\text{exp}\{\frac{\beta \mu }{1-\mu }\}-1}{\beta }+\frac{\mu }{1-\mu }\text{log}{\left(1-\tau \right)}^{-1}\sum_{i=1}^{n}\text{exp}\{\frac{\beta \;y_{i}}{1-y_{i}}\}\\ & +\text{log}{\left(1-\tau \right)}^{-1}\sum_{i=1}^{n}\frac{y_{i}}{1-y_{i}}\;\text{exp}\{\frac{\beta y_{i}}{1-y_{i}}\}, \end{array}$$

and

$$\begin{array}{cc} U_{\mu } & =\frac{\partial \ell }{\partial \mu }=\frac{\text{log}[\text{log}{\left(1-\tau \right)}^{-1}]}{{\left[\text{exp}\{\frac{\beta \;\mu }{1-\mu }\}-1\right]}^{2}}\sum_{i=1}^{n}\text{exp}\{\frac{\beta y_{i}}{1-y_{i}}\}-n\;-\frac{\text{exp}\{\frac{\beta \;\mu }{1-\mu }\}}{\text{exp}\{\frac{\beta \;\mu }{1-\mu }\}-1}\\ & -n\;\text{log}[\text{log}{\left(1-\tau \right)}^{-1}]. \end{array}$$

Note that the system of equations *U*_***θ***_ = **0** cannot be solved in closed form; therefore, the maximization of (16) to obtain the EMV of ***θ*** can be carried out using the quasi-Newton BFGS nonlinear optimization algorithm implemented in the optim function available in R.

## 5 Simulation study

In this Section, a Monte Carlo study is carried on to evaluate the performance of the MLEs of the UREW family in finite samples. For that, the URR and URG distributions are considered. This study conducted 10,000 Monte Carlo replications with sample sizes *n* ∈ {10, 25, 50, 75, 100}. Aiming to evaluate the point estimators, we use the set of estimates of parameters obtained in each replication to calculate its mean, variance, relative biases (RBs), standard deviations (SDs), and root mean squared errors (RMSEs). Regarding the initial values selected for simulation, we highlight that the URR distribution has a closed form for its MLE (see Eq (14)). Therefore, one advantage of using this model is that it does not require defining initial values in the ML method. For the two-parameter UREW special cases, we use the Broyden-Fletcher-Goldfarb-Shanno (BFGS) algorithm and compute the observed information matrix numerically from the optim function in the R programming language. Therefore, we set the sample quantile as the initial value for *μ* and one for the shape parameter. These values are used either for the simulated or actual data experiments performed in the paper. We calculate the coverage probability of the 95% pointwise confidence interval (CP_95%_) to evaluate the interval estimation. Next, we provide the numerical results for both considered distributions. Next, the numerical results for both distributions considered are presented.

### 5.1 Numerical Analysis for the URR distribution

We generate occurrences of the variable *Y* following a URR law with five different values of *μ* (scenarios). For that, we use the inversion method replacing *u* ∼ *U*(0, 1) in the URR qf. The simulation results are shown in Table 2. It reveals low RB values in all the scenarios and sample sizes considered. We highlight that all its observed values are less than 0.7%. We also observe low SD values, all less than 0.5. For all the sample sizes, it is common to observe RMSE’s lower values for the central values of *μ* (*μ* = 0.4, for example) than for the close values of the extremes (*μ* = 0.15 or *μ* = 0.9, for example). In its last column, it can verify that the coverage probabilities of the 95% pointwise confidence intervals of the parameter are quite close to the nominal level.

**Table 2:** Results of the Monte Carlo simulation from the URR distribution.

Scenario	*μ*	*n*	mean	Variance	RB %	RMSE	CP_95%_
1	0.15	10	0.1475	0.0004	−0.2432	0.0003	0.9490
		25	0.1492	0.0001	−0.0741	0.0001	0.9430
		50	0.1493	0.0001	−0.0617	0.0001	0.9590
		75	0.1494	0.0001	−0.0505	0.0001	0.9480
		100	0.1497	0.0001	−0.0286	0.0001	0.9580
2	0.25	10	0.2463	0.0008	−0.3669	0.0009	0.9470
		25	0.2484	0.0003	−0.1584	0.0003	0.9579
		50	0.2492	0.0001	−0.0773	0.0001	0.9533
		75	0.2495	0.0001	−0.0446	0.0001	0.9537
		100	0.2497	0.0001	−0.0287	0.0001	0.9518
3	0.4	10	0.3943	0.0014	−0.5635	0.0015	0.9552
		25	0.3976	0.0005	−0.2385	0.0005	0.9594
		50	0.3988	0.0002	−0.1170	0.0002	0.9538
		75	0.3993	0.0002	−0.0689	0.0001	0.9536
		100	0.3995	0.0001	−0.0459	0.0001	0.9508
4	0.7	10	0.6933	0.0012	−0.6654	0.0012	0.9518
		25	0.6972	0.0004	−0.2725	0.0004	0.9535
		50	0.6986	0.0002	−0.1341	0.0002	0.9506
		75	0.6991	0.0001	−0.0812	0.0001	0.9521
		100	0.6994	0.0001	−0.0562	0.0001	0.9469
5	0.9	10	0.8966	0.0002	−0.3384	0.0002	0.9405
		25	0.8986	0.0001	−0.1356	0.0001	0.9489
		50	0.8993	0.0001	−0.0666	0.0001	0.9497
		75	0.8995	0.0001	−0.0408	0.0001	0.9515
		100	0.8997	0.0001	−0.0287	0.0001	0.9454

**Fig 2:**
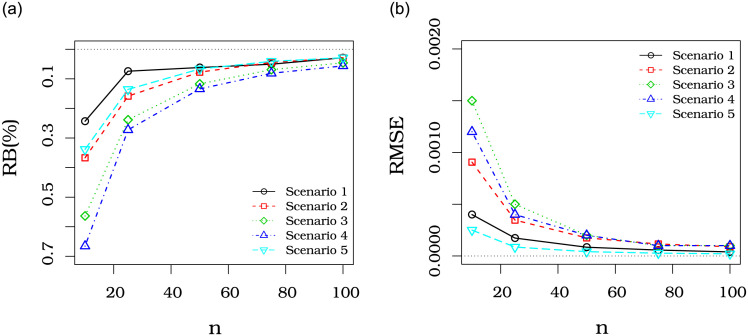
Percentual RB and RMSE of the URR estimator in several scenarios.

Fig 2 indicates that the RB and RMSE of $\hat{\mu }$ decrease as the sample size increases, corroborating the asymptotic properties of the MLEs.

### 5.2 Numerical analysis for the URG distribution

Analogous to the previous experiment, occurrences of the variable *Y* are initially generated, which follows a URG distribution with different configurations in its parameters *μ* and *β*. The data are generated using the inversion method in the URG qf. In Table 3‘, we present the simulation results. It shows that *μ*’s estimates are more accurate than *β*’s. We can also observe that the RB of *μ* is always less than 0.4% in absolute value. For sample sizes greater than 75, the RB of $\hat{\beta }$ is always less than 10%. In the last column of Table 2, we can be observed that the coverage probabilities of the 95% pointwise confidence intervals of both parameters are quite close to the nominal level.

**Table 3:** Results of the Monte Carlo simulation from the URG distribution.

Scenario	*μ*	*β*	*n*	Mean		Variance		RB %($\hat{\mu }$)		RMSE($\hat{\mu }$)		CP_95%_	
				$\hat{\mu }$	$\hat{\beta }$	$\hat{\mu }$	$\hat{\beta }$	$\hat{\mu }$	$\hat{\beta }$	$\hat{\mu }$	$\hat{\beta }$	$\hat{\mu }$	$\hat{\beta }$
1	0.4	0.8	10	0.4038	1.2889	0.0046	0.7510	0.3819	48.8994	0.0047	0.9902	0.8852	0.9311
			25	0.4014	0.9674	0.0019	0.1895	0.1444	16.7461	0.0019	0.2176	0.9268	0.9433
			50	0.4011	0.8856	0.0010	0.0827	0.1146	8.5658	0.0010	0.0901	0.9377	0.9474
			75	0.4009	0.8571	0.0006	0.0526	0.0995	5.7184	0.0006	0.0559	0.9410	0.9489
			100	0.4006	0.8400	0.0005	0.0384	0.0653	4.0097	0.0005	0.0400	0.9423	0.9462
2	0.5	1.5	10	0.4971	1.9084	0.0025	0.6566	−0.2869	40.8414	0.0025	0.8234	0.8941	0.9427
			25	0.4985	1.6408	0.0010	0.1832	−0.1444	14.0812	0.0010	0.2031	0.9283	0.9480
			50	0.4994	1.5709	0.0004	0.0824	−0.0544	7.0981	0.0004	0.0874	0.9406	0.9499
			75	0.4998	1.5485	0.0003	0.0534	−0.0195	4.8538	0.0003	0.0558	0.9412	0.9498
			100	0.4997	1.5333	0.0002	0.0393	−0.0226	3.3385	0.0002	0.0404	0.9444	0.9476
3	0.6	2.8	10	0.5980	3.2892	0.0004	1.0709	−0.1989	48.9285	0.0004	1.3103	0.9042	0.9526
			25	0.5991	2.9682	0.0001	0.2841	−0.0875	16.8275	0.0001	0.3124	0.9339	0.9508
			50	0.5996	2.8829	0.0008	0.1265	−0.0388	8.2924	0.0008	0.1333	0.9430	0.9513
			75	0.5998	2.8571	0.0001	0.0816	−0.0182	5.7180	0.0001	0.0849	0.9437	0.9510
			100	0.5998	2.8390	0.0001	0.0601	−0.0167	3.9059	0.0001	0.0616	0.9456	0.9475
4	0.7	5	10	0.6996	5.8574	0.0001	3.0886	−0.0362	85.7439	0.0001	3.8238	0.9016	0.9499
			25	0.6998	5.2956	0.0001	0.7867	−0.0164	29.5603	0.0001	0.8741	0.9350	0.9507
			50	0.6999	5.1454	0.0001	0.3466	−0.0066	14.5412	0.0001	0.3677	0.9436	0.9504
			75	0.6999	5.0996	0.0001	0.2220	−0.0018	9.9651	0.0001	0.2319	0.9435	0.9505
			100	0.6999	5.0688	0.0001	0.1630	−0.0022	6.8801	0.0001	0.1677	0.9449	0.9481
5	0.7	0.9	10	0.6961	1.1001	0.0012	0.1686	−0.3809	20.0002	0.0012	0.2086	0.8990	0.9485
			25	0.6983	0.9690	0.0004	0.0471	−0.1693	6.9054	0.0004	0.0519	0.9297	0.9507
			50	0.6992	0.9345	0.0002	0.0212	−0.0738	3.4534	0.0002	0.0224	0.9420	0.9513
			75	0.6996	0.9238	0.0001	0.0138	−0.0374	2.3819	0.0001	0.0143	0.9415	0.9507
			100	0.6996	0.9162	0.0001	0.0101	−0.0337	1.6285	0.0001	0.0104	0.9446	0.9486
6	0.75	1.1	10	0.7475	1.3025	0.0004	0.1828	−0.2473	20.2578	0.0004	0.2239	0.9037	0.9520
			25	0.7489	1.1697	0.0001	0.0496	−0.1068	6.9781	0.0001	0.0545	0.9330	0.9515
			50	0.7495	1.1345	0.0001	0.0222	−0.0477	3.4546	0.0001	0.0234	0.9432	0.9516
			75	0.7497	1.1238	0.0001	0.0144	−0.0244	2.3864	0.0001	0.0149	0.9429	0.9515
			100	0.7497	1.1163	0.0001	0.0106	−0.0214	1.6317	0.0001	0.0108	0.9452	0.9470

**Fig 3:**
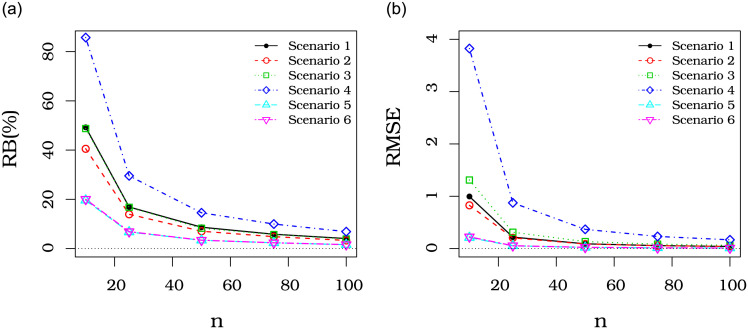
Total percentual RB and total RMSE of the URG estimators in several scenarios. (a) VR(%) total, (b) EQM total.

Fig 3(a) presents a plot with the sum of the RB of $\hat{\mu }$ and $\hat{\beta }$ that we call the total RB. Fig 3(b) presents a similar plot with the sum of the RMSE of $\hat{\mu }$ and $\hat{\beta }$, that we call the total RMSE. They show that the total RB and total RMSE of $\hat{\mu }$ and $\hat{\beta }$ decrease as the sample size increases, corroborating the asymptotic properties of the MLEs.

## 6 Applications

This section illustrates the usefulness of the UREW family through applications in educational data related to student dropout, also known as student attrition. This outcome has some complexity in data collection [42], and a diversity of definitions has been considered in the specialized literature. In this paper, we are interested in analyzing the first-year dropout rate in undergraduate courses, defined as the proportion of students who withdraw from the course before completing the first year. Thus, from a sample with *n* undergraduate courses, the *i*th observation is obtained as

$$\begin{array}{c} {\text{DROPOUT\_RATE}}_{i}=\frac{\begin{array}{c} \text{number}\;\text{of}\;\text{freshmen}\;\text{students}\;\text{who}\;\text{dropped}\;\text{out}\\ \text{the}\;i\text{th}\;\text{course}\;\text{before}\;\text{completing}\;\text{the}\;\text{first}\;\text{year} \end{array}}{\text{number}\;\text{of}\;\text{freshmen}\;\text{students}\;\text{enrolled}\;\text{in}\;\text{the}\;i\text{th}\;\text{course}}, \end{array}$$

where *i* ∈ {1, …, *n*}. The decision to focus the study on freshmen students lies in the evidence that the risk of dropping out is higher during the first year of college, also called the freshmen year [42, 43]. This period is seen as the most critical time for the connection between academic programs and students [44]. Therefore, understanding the behavior of this variable may be helpful in developing practices aimed at reducing the early dropout from undergraduate courses from different areas.

The data used in this case study were collected from the Brazilian higher education census microdata, conducted in 2018 [45] and were calculated from the entering students in 2018. We select the presential courses with more than 29 new students and first-year dropout rate in the (0, 1) interval in the census academic year. The applications refer to four data sets about civil engineering, economics, computer sciences, and control engineering courses. We fit UREW special models and compare their performance with other existing double-bounded distributions, which are not special cases of the proposed family.

Table 4 gives a descriptive summary of the dropout rates of each dataset considered. The Economics course exhibits smaller values for all central tendency measures and higher for the skewness, kurtosis, and amplitude measures. The other courses present those measures quite close when compared with each other. Their mean and median are around 17% and 16%, respectively. The descriptive measures indicate that, for all data sets, the mass of observations concentrates on the left. This configuration is adequate since the dropout rate is negatively related to institutional quality and effectiveness. Academic programs with low dropout rates are often considered to be more efficient [34]. Nevertheless, the dropout rates in higher education are social and institutional concerns [42], and there is a broad consensus on the need for universities to promote students’ success [46]. The fact that many students do not achieve their goals during university experience is a waste of talent and human potential [42, 46].

**Table 4:** Descriptive statistics for the dropout rates in the four course types considered.

Course type	Mean	Median	Variance	Skewness	Kurtosis	Min.	Max.	n
Civil engineering	0.1781	0.1667	0.0143	0.6219	-0.2310	0.0052	0.5750	658
Economics	0.1260	0.1140	0.0105	2.0595	8.3647	0.0083	0.7381	132
Computer Sciences	0.1702	0.1528	0.0139	0.8284	0.5582	0.0085	0.6410	255
Control engineering	0.1715	0.1667	0.0133	0.6833	-0.1512	0.0132	0.4884	97

For modeling these data, we fit five UREW special models studied in the current paper, i.e., the URG,URBXII,URL,URR, and URW distributions. Their densities are given by equations (5), (7), (9), (11), and (10), respectively. We fix *τ* at 0.5 in those equations. We also considered six well-known alternative distributions to describe random variables supported in the unit interval for comparison purposes. We fit the Beta, KW, UBS, UG, UW, and complementary unit Weibull (CUW) [14] distributions. They do not represent UREW special cases and are selected as competitive distributions due to their relevance in the literature. The beta and KW are classical models for double-bounded outcomes. The UG is chosen due to its relevance to various problems. It has received a great deal of attention from statisticians for developing methodological advances [47]. The UBS and UW are two of the most relevant models regarding recent advances in distribution theory. The CUW arises as an alternative model due to its usefulness regarding educational modeling. This distribution has proved helpful in analyzing literacy rates [14]. The densities of all these competitor models are presented in Appendix A.

Parameter estimation is performed by the maximum likelihood method for all fitted models, and the Cramér-von Misses corrected statistic [48] (W*) is considered as the goodness-of-fit measure. Those estimates are computed using the goodness.fit function from the AdequacyModel package [49]. The goodness.fit function allows computing the MLEs of probability distributions and their goodness-of-fit statistical measures. It uses the optim function in the implementation and includes several optimization techniques. For the paper results, we use the BFGS algorithm and compute the observed information matrix numerically. Thus, the standard errors and confidence intervals were obtained from the asymptotic normality property of the MLEs. We set the initial values at 1 for the shape (or precision) parameter, the sample mean for the distributions indexed in the mean, and the sample quantile for those with quantile parametrization.

**Fig 4:**
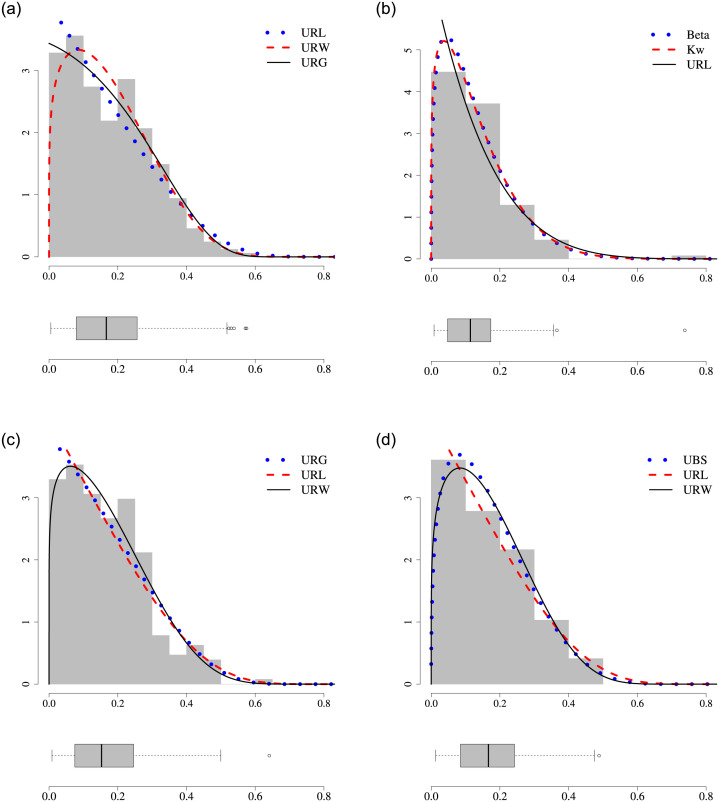
Histogram and estimated densities for the applications.

The estimation results for all data sets are reported in Table 5. We observe that the distributions on the UREW family have the lowest W* for the course types considered. The proposed models occupy the first three positions in the ranking for civil engineering and computer sciences. Analyzing the control engineering course, we note that the URW outperforms the others and is followed by the URL distribution, which also belongs to the UREW family. For the economics course, the URL distribution has superior goodness-of-fit. Fig 4 displays the boxplots and the histograms with fitted density functions for the three best models according to W*. Those plots corroborate that the UREW fits are adequate to the dropout rates of all course types considered and provides real improvement over existing distributions. Therefore, the proposed family is shown competitive with classical unit models such as the beta and KW distributions.

**Table 5:** MLEs estimates, the corresponding standard errors (given in parentheses) and goodness-of-fit measure for all fitted models and course types considered.

**Distributions**	**Civil engineering**				**Economics**			
	**Estimates**		W*	**Ranking**	**Estimates**		W*	**Ranking**
Beta(*φ*, *μ*)	8.7209	0.1771	0.4402	9	10.8025	0.1278	0.0691	2
	(0.4763)	(0.0048)			(1.3524)	(0.0084)		
KW(ϕ,μ)	1.3856	0.1553	0.3650	8	1.2090	0.1052	0.0706	3
	(0.0494)	(0.0049)			(0.0895)	(0.0085)		
UG(ϕ,μ)	5.569	0.1771	0.5817	10	6.8278	0.1265	0.0787	7
	(0.2983)	(0.0049)			(0.8207)	(0.0087)		
UBS(β,μ)	0.4411	1.8501	0.2562	4	0.4238	2.2141	0.0877	8
	(0.0122)	(0.0310)			(0.0261)	(0.0798)		
UW(β,μ)	2.4602	0.1382	1.3007	11	2.8739	0.0920	0.1929	10
	(0.0719)	(0.0051)			(0.1902)	(0.0078)		
CUW(β,μ)	1.3224	0.1572	0.3128	5	1.1478	0.1043	0.0712	5
	(0.0409)	(0.0050)			(0.0723)	(0.0088)		
URBXII(β,μ)	1.2876	0.1576	0.3167	6	1.1415	0.1049	0.072	6
	(0.0364)	(0.0050)			(0.0670)	(0.0088)		
URG(β,μ)	0.7173	0.1586	0.1719	1	0.7471	0.0947	0.0710	4
	(0.1731)	(0.0059)			(0.3012)	(0.0084)		
URL(β,μ)	50.2648	0.1456	0.2462	3	1.111	0.0955	0.0687	1
	(54.5751)	(0.0049)			(0.6462)	(0.0087)		
URR(μ)	0.2133		0.3412	7	0.2074		0.3673	11
	(0.0033)				(0.0072)			
URW(β,μ)	1.1797	0.1609	0.2117	2	0.9861	0.1047	0.0936	9
	(0.0358)	(0.0053)			(0.0585)	(0.0096)		
**Distributions**	**Computer sciences**				**Control engineering**			
	**Estimates**		W*	**Ranking**	**Estimates**		W*	**Ranking**
Beta(*φ*, *μ*)	8.689	0.1695	0.1371	8	9.1876	0.1706	0.0494	8
	(0.7654)	(0.0075)			(1.3106)	(0.0119)		
KW(ϕ,μ)	1.3346	0.1466	0.1038	7	1.3969	0.1496	0.0371	7
	(0.0761)	(0.0076)			(0.1285)	(0.0121)		
UG(ϕ,μ)	5.5231	0.1695	0.215	10	5.8359	0.1707	0.0756	10
	(0.4751)	(0.0078)			(0.8152)	(0.0124)		
UBS(β,α)	0.4455	1.9038	0.071	4	0.4299	1.8902	0.0294	3
	(0.0197)	(0.0518)			(0.0309)	(0.0806)		
UW(β,μ)	2.4404	0.1304	0.5647	11	2.5101	0.133	0.2082	11
	(0.1141)	(0.0080)			(0.1906)	(0.0128)		
CUW(β,μ)	1.2758	0.148	0.0857	6	1.3319	0.1514	0.0315	5
	(0.0626)	(0.0079)			(0.1068)	(0.0125)		
URBXII(β,μ)	1.2489	0.1485	0.0833	5	1.2956	0.1518	0.0309	4
	(0.0561)	(0.0079)			(0.0949)	(0.0126)		
URG(β,μ)	0.2618	0.1446	0.0564	2	0.8688	0.1532	0.0318	6
	(0.2490)	(0.0088)			(0.4991)	(0.0148)		
URL(β,μ)	28.8352	0.1396	0.0673	3	33.445	0.1394	0.0282	2
	(51.2034)	(0.0076)			(76.399)	(0.0123)		
URR(μ)	0.2111		0.1705	9	0.2038		0.0676	9
	(0.0052)				(0.0082)			
URW(β,μ)	1.1321	0.1512	0.0561	1	1.1936	0.1547	0.0258	1
	(0.0541)	(0.0083)			(0.0942)	(0.0131)		

The UREW special cases also exhibit superior performance when compared to recent alternatives, including the UBS, UW, and CUW distributions. It is worth noting that the CUW distribution has been commonly used in educational modeling. In [14], it was verified that this model can properly fit literacy rates. However, it is important to highlight that while higher literacy rates are desirable [14], lower values are considered more favorable in the case of dropout rates [17]. In this case, it is expected that left-skewed distributions to fit better the former and right-skewed distributions to be more suitable for the latter. This feature may explain why the CUW is not among the best models for the analyzed datasets while evincing the capacity of the UREW family to model the first-year dropout rate effectively.

Our results may represent useful tools for universities to evaluate and improve their programs. It is a relevant application as it allows us to deal with the academic, social, and economic implications of university dropout [17]. Nevertheless, other potential applications can be explored in the context of educational modeling. The new family can be competitive to model literacy rates [14], educational attainment percentages [31], proportions of adolescents who want top grades at school [32], and proportions of the novice teachers with a mentor at the school [33]. These variables have been explored through other commonly used distributions in educational modeling. We can also cite the graduation and persistence rates as further applications, which are related to student progression and academic success patterns [34].

## 7 Final remarks

This paper defines the unit ratio-extended Weibull (UREW) family of distributions. It is obtained on a ratio transformation in the extended Weibull family and can be used to model continuous random variables in the unit interval. The new family has a closed-form for quantile measures; thus, we provide a quantile parametrization for the family. Several special cases are derived, and parameter estimation is explored using the maximum likelihood theory. We show that some one-parameter UREW special cases may present closed-form for the maximum likelihood estimator (MLE). We perform Monte Carlo experiments to assess the performance of those estimators. For example, the unit ratio-Rayleigh MLE is approximately unbiased for small sample sizes. We also note an appropriate performance for the unit ratio-Gompertz MLEs. The utility of the proposed family is illustrated with applications to the first-year dropout rate of undergraduate courses in Brazilian universities. We select four course types and note that, for those data, the UREW special models fit properly and outperform other classical and recent unit distributions. Thus, the new family can be competitive alternative when those models are unsuitable. We emphasize that a long list of possibilities can be addressed in future works. For example, our approach can be investigated in the presence of zeros and ones, and quantile regression models are also a natural path. The UREW can also be generalized to accommodate time-dependent double-bounded indicators by using the autoregressive moving average models. This kind of structure is in the state-of-art literature on the analysis of double-bounded time series. The UREW can also attract applications to other double-bounded variables, being a competitive option to other unit distributions commonly used in educational modeling. For instance, literacy rates, educational attainment percentages, graduation, and persistence rates are educational measurements that represent potential applications for the proposed family.

## Appendix

### A—Alternative distributions fitted in the applications

In this appendix, we present the unit distributions fitted in Section 6 as alternative models to the UREW family. These model and their corresponding densities are listed bellow (for 0 < *y* < 1):

- The beta density is given by
  $$\begin{array}{c} f_{\text{Beta}}\left(y\right)=\frac{\Gamma (\phi )}{\Gamma (\mu \;\phi )\Gamma ((1-\mu )\phi )}\;y^{\mu \;\phi -1}\;{\left(1-y\right)}^{(1-\mu )\phi -1}, \end{array}$$
  where *μ* ∈ (0, 1) is the mean of *Y* and *ϕ* > 0 is a precision parameter. The above parametrization is pioneered by [50].
- The KW density is given by
  $$\begin{array}{c} f_{KW}\left(y\right)=\frac{\phi \;\text{log}(1-q)}{\text{log}(1-e^{-\phi /\text{log}(\mu )})}\;y^{-\phi /\text{log}(\mu )-1}\;{\left(1-y^{-\phi /\text{log}(\mu )}\right)}^{\text{log}\left(1-q\right)/\text{log}\left(1-e^{-\phi }\right)-1}, \end{array}$$
  where *μ* ∈ (0, 1) is the *q*th quantile parameter, and *ϕ* > 0 is a precision parameter. The above parametrization is pioneered by [51]. In Section 6 we fix *q* at 0.5 thus the parameter *μ* refers to the median of *Y*.
- The UG density is given by
  $$\begin{array}{c} f_{UG}\left(y\right)=[\frac{\mu^{1/\phi }}{1-\mu^{1/\phi }}{]}^{\phi }\;\;\frac{1}{\Gamma (\phi )}\;y^{\mu^{1/\phi }/\left[\left(1-\mu^{1/\phi }\right)-1\right]}\;{\left[-\text{log}\left(y\right)\right]}^{\phi -1}, \end{array}$$
  where *μ* ∈ (0, 1) is the mean of *Y* and *ϕ* > 0 is a precision parameter. The above parametrization is pioneered by [52].
- The UBS density is given by
  $$\begin{array}{cc} f_{UBS}\left(y\right) & =\frac{1}{2\;y\;\alpha \;\beta \;\sqrt{2\;\pi }}[{\left(-\frac{\beta }{\text{log}(y)}\right)}^{\frac{1}{2}}+{\left(-\frac{\beta }{\text{log}(y)}\right)}^{\frac{3}{2}}]\\ & \times \text{exp}\{\frac{1}{2\;\alpha^{2}}\;(\frac{\text{log}(y)}{\beta }+\frac{\beta }{\text{log}(y)}+2)\}, \end{array}$$
  where *α* > 0 and *β* > 0 are shape parameters. The UBS is pioneered by [11].
- The UW density is given by
  $$\begin{array}{c} f_{UW}\left(y\right)=\frac{\beta }{y}(\frac{\text{log}\;\tau }{\text{log}\;\mu })(\frac{\text{log}\;y}{\text{log}\;\mu }{)}^{\beta -1}\tau^{{\left(\text{log}\;y/\text{log}\;\mu \right)}^{\beta }}, \end{array}$$
  where *μ* ∈ (0, 1) is the *τ*th quantile parameter and *β* > 0 are shape parameters. The above parametrization is pioneered by [41]. In Section 6 we fix *τ* at 0.5 thus the parameter *μ* refers to the median of *Y*.
- The CUW density is given by
  $$\begin{array}{c} f_{CUW}\left(y\right)=\frac{\beta \;\text{log}\;2}{1-y}{\left[-\text{log}\left(1-\mu \right)\right]}^{-\beta }\;[-\text{log}(1-y){]}^{\beta -1}\;2^{-{\left[\text{log}\left(1-y\right)/\text{log}\left(1-\mu \right)\right]}^{\beta }}, \end{array}$$
  where *μ* ∈ (0, 1) is the median of *Y* and *β* > 0 is a shape parameter. The above distribution is pioneered by [14].

## Supporting information

S1 Data (ZIP)
